# Optical Image Sensors for Smart Analytical Chemiluminescence Biosensors

**DOI:** 10.3390/bioengineering11090912

**Published:** 2024-09-12

**Authors:** Reza Abbasi, Xinyue Hu, Alain Zhang, Isabelle Dummer, Sebastian Wachsmann-Hogiu

**Affiliations:** Department of Bioengineering, McGill University, Montreal, QC H3A 0E9, Canada; reza.abbasi@mail.mcgill.ca (R.A.); xinyue.hu@mail.mcgill.ca (X.H.); alain.zhang@mail.mcgill.ca (A.Z.); isabelle.dummer@mail.mcgill.ca (I.D.)

**Keywords:** biosensor, optical, chemiluminescence, image sensors, machine learning

## Abstract

Optical biosensors have emerged as a powerful tool in analytical biochemistry, offering high sensitivity and specificity in the detection of various biomolecules. This article explores the advancements in the integration of optical biosensors with microfluidic technologies, creating lab-on-a-chip (LOC) platforms that enable rapid, efficient, and miniaturized analysis at the point of need. These LOC platforms leverage optical phenomena such as chemiluminescence and electrochemiluminescence to achieve real-time detection and quantification of analytes, making them ideal for applications in medical diagnostics, environmental monitoring, and food safety. Various optical detectors used for detecting chemiluminescence are reviewed, including single-point detectors such as photomultiplier tubes (PMT) and avalanche photodiodes (APD), and pixelated detectors such as charge-coupled devices (CCD) and complementary metal–oxide–semiconductor (CMOS) sensors. A significant advancement discussed in this review is the integration of optical biosensors with pixelated image sensors, particularly CMOS image sensors. These sensors provide numerous advantages over traditional single-point detectors, including high-resolution imaging, spatially resolved measurements, and the ability to simultaneously detect multiple analytes. Their compact size, low power consumption, and cost-effectiveness further enhance their suitability for portable and point-of-care diagnostic devices. In the future, the integration of machine learning algorithms with these technologies promises to enhance data analysis and interpretation, driving the development of more sophisticated, efficient, and accessible diagnostic tools for diverse applications.

## 1. Introduction

Biosensors are analytical devices that use biological components to detect, convert, quantify, and report biological information, such as metabolite concentration. They are essential tools for diagnosing and monitoring health conditions such as infectious diseases [1,2,3,4] and glucose levels [5,6,7], as well as environmental sensing for chemical pollution [8,9,10] and microbial contamination [11,12,13,14]. Biosensors with point-of-care (POC) and point-of-need (PON) capability address spatial and temporal constraints of sample handling by offering portability and rapid results, making these devices suitable for use in field settings, including at home, at bedside, and during clinical appointments [15]. Given their use case scenarios, key criteria for POC and PON biosensors include sensitivity, specificity, portability, and fast result turnover.

Transducers in biosensors convert the binding events between the target analyte and the biorecognition element into various types of signals, including electrochemical, optical, mechanical, temperature, electric, or magnetic signals [16,17]. Biosensors can be categorized based on the type of signals the transducer produces, with electrochemical and optical biosensors being the most commonly available biosensors translated from laboratory to market due to their high sensitivity and ease of integration with miniaturized, on-chip systems. Various articles have reviewed the topic of biosensors, offering comprehensive overviews that describe their components, mechanisms, historical development, current trends towards miniaturization and wearability, and applications across various fields, including medical diagnosis and environmental monitoring [17,18,19]. In this paper, we will focus on optical biosensors due to their superior signal-to-noise ratio and visual readout capability.

Biosensors that utilize an optical transducing mechanism, commonly known as optical biosensors, are the most prevalent type of biosensor. The optical transducer converts binding events between the target analyte and biorecognition element into optical signals such as light emission or spectral change. Optical biosensors offer several advantages across a broad range of applications, particularly due to their high sensitivity, high specificity, reliability, versatility, and real-time detection capabilities [20,21,22,23]. As highly sensitive and easy-to-use devices, optical biosensors have been extensively applied in research, particularly as lab-on-chip devices in drug discovery and in the characterization of biomolecular interactions [24,25]. They are also prominent in POC and PON applications, including pathogen detection for clinical diagnostics, on-site pollution detection for environmental monitoring, and contaminant detection in food [26,27,28].

Optical biosensors can be categorized into three main types: colorimetric, luminescent, and refractive index-based, each with distinct signal generation mechanisms. Colorimetric biosensors detect changes resulting from reflection, absorption, or scattering of light, often involving coupling with chromogenic indicators or nanoparticle agglutination that produce visible color changes in the presence of a target analyte [29,30]. Luminescent biosensors measure light emitted from analyte-specific biochemical reactions that cause excited molecules to emit light as they return to the ground state. Refractive index-based biosensors detect changes in the refractive index of samples using plasmonic, resonance, or interference methods [31].

Luminescence-based biosensors offer significant advantages due to their higher signal-to-noise ratio, which enhances the sensitivity of biosensors. Therefore, luminescence-based biosensors are particularly useful in applications requiring high sensitivity and low background interference. Their ability to detect analytes at low concentrations makes luminescence-based biosensors highly valuable in clinical diagnostics and environmental monitoring. Luminescence can be generated through fluorescence, chemiluminescence (CL), bioluminescence (BL), and electrochemiluminescence (ECL). Fluorescence-based biosensors rely on the emission of light from fluorophores when excited by a specific wavelength of light [32]. In chemiluminescence-based biosensors, detection involves the light generated from chemical reactions that induce an excited state in a species [33]. Bioluminescence is a special type of chemiluminescence that occurs within biological systems, such as those involving luciferin and luciferase. Electrochemiluminescence, on the other hand, is chemiluminescence induced by an electrical potential. Among luminescence-based biosensors, chemiluminescence, bioluminescence, and electrochemiluminescence are particularly advantageous as they bypass the need for external illumination, resulting in simpler systems with even higher signal-to-noise ratios. ECL further stands out from bioluminescence and chemiluminescence as the timing and intensity of its reactions can be controlled by the applied electrical potential, enabling temporal and quantitative control of the reaction.

To measure the optical signal produced during analyte-specific reactions, optical detectors are an integral part of an optical biosensor. Optical detectors in luminescence-based biosensors function by converting light to electrical signals. These detectors can be point detectors such as photomultiplier tubes (PMTs), which use photosensitive tubes with dynodes to transfer and exponentially amplify electronic signals through cascading secondary emissions. Multiple PMTs can be arranged in multiplex configurations to create a plate reader with higher throughput. Although PMTs are sensitive and low-noise, they are not ideal for POC applications due to their delicate materials, high voltage requirements, and high cost. Alternatively, avalanche photodiodes (APDs) use doped semiconductive materials to convert photons into electrons, which are then amplified through impact ionization. Arrays of APDs can be used to build image sensors, such as complementary metal–oxide–semiconductor (CMOS) image sensors, which offer multiplexity and spatial resolution. While APDs generally have higher noise levels than PMTs, they are sensitive, high-speed, inexpensive, and compact. Therefore, image sensors are ideal for POC and PON optical detection.

While image sensors are traditionally coupled with lenses to adjust their focal distance, field of view (FOV), and spatial resolution, the lenses introduce a trade-off between FOV and spatial resolution. On the other hand, using image sensors without lenses eliminates this trade-off, as the FOV will be determined by the number and size of the pixels in the sensor, and the spatial resolution will be determined by the size of the pixels. With the absence of lenses, the distance between the sample and the image sensor must be minimized to obtain a clear, sharp image and avoid diffraction patterns. This configuration, known as lensless contact imaging, maximizes photon collection efficiency due to the close proximity between the sample and sensor and is ideal for luminescence-based biosensors [34]. Fluorescence imaging requires an emission filter to be positioned between the sample and the sensor to eliminate excitation wavelengths, complicating its implementation in a lensless contact setup. Therefore, luminescence-based biosensors in lensless contact configurations commonly utilize CL, ECL, and BL as signal generation methods. To facilitate more precise as well as spatially and temporally controlled sample handling, lensless contact imaging is often integrated with microfluidic components. Additionally, microfluidic components enable multiplexity by introducing multiple channels. These advantages favor the development of microfluidic-integrated lensless image-sensing configurations in optical biosensors, which will be discussed in this paper. As the spatial resolution of a lensless image sensor in contact mode is determined by the pixel size, which is typically a few micrometers but can be submicrometer, lensless contact imaging has been used as a compact and inexpensive microscope in POC or PON settings for cell imaging. Moreover, a microfluidic-integrated lensless image sensor with a luminescence-based analyte detection technique is also used in various biosensing applications beyond its traditional imaging roles.

In this paper, we focus on chemiluminescence-based biosensors integrated with lensless contact-mode image sensors for POC and PON applications, highlighting the integration of such systems with microfluidic components and artificial intelligence. We begin by discussing the fundamental principles of signal generation in chemiluminescence-based biosensors and the optical detection technologies used in these biosensors. We then explore the integration of image sensors with luminescence-based biosensing technologies, microfluidic sample handling methodologies, and artificial intelligence. Finally, we discuss the applications of image-based biosensors at the POC and PON, providing an outlook for the field of optical biosensing.

Several reviews have discussed the recent advancements in optical biosensors, covering the fabrication, application, historical overview, and strengths and weaknesses of various types of optical sensors, as well as the integration of optical biosensors with microfluidic channels for point-of-care testing [20,21,35,36]. These articles mainly present fundamental principles and applications, lacking a focused perspective. Our paper offers an outlook and road map for the development of smart optical biosensors by specifically exploring chemiluminescence-based optical biosensors, not only detailing the mechanisms of signal generation and detection but also examining the integration of optical biosensors with microfluidic systems for sample handling, image sensors for readout, and machine learning for signal processing and interpretation.

## 2. Mechanisms of Signal Generation for Chemiluminescence Biosensors

**Figure 1:**
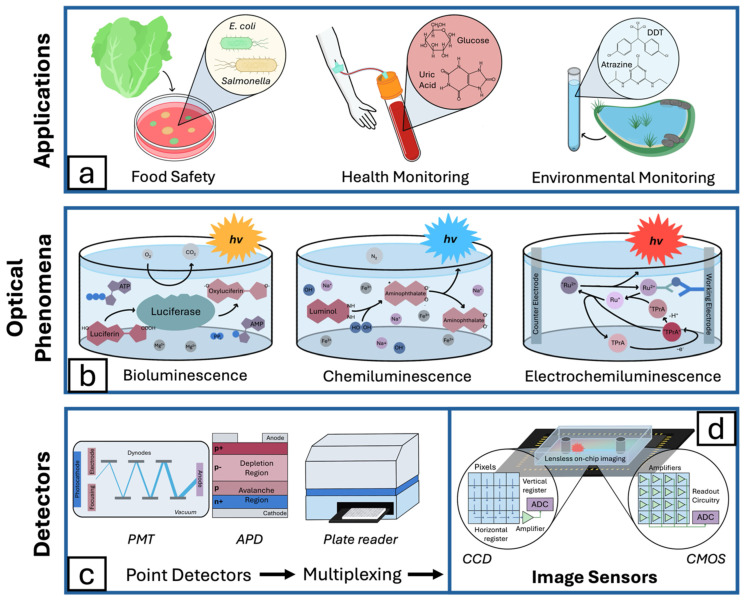
Overview of the analytical chemiluminescence sensors: (**a**) application of analytical chemiluminescence in food safety, health monitoring, and environmental monitoring; (**b**) optical phenomena including bioluminescence, chemiluminescence, and electrochemiluminescence; (**c**) optical detectors from point detectors (left) including photomultiplier tub and avalanche photodiode to multiplexing (plate reader) (**d**) optical image sensors including CCD and CMOS image sensors.

Luminescence-based optical biosensors offer several significant advantages, including contactless detection, high sensitivity, and the ability to detect a wide range of analytes through labeling [37]. These biosensors are suitable for a diverse array of applications (Figure 1a), such as medical diagnostics, environmental monitoring, food safety, drug discovery, and biomedical research. Chemiluminescence and electrochemiluminescence (Figure 1b), which simplify signal transduction, provide means of producing luminescence without the need for excitation light. Both methods rely on chemical reactions to generate light, with their differences leading to notable variations in sensor design, performance, and application. Each method has distinct advantages and drawbacks, contributing to their respective suitability for specific applications.

### 2.1. Chemiluminescence

The mechanisms underlying chemiluminescence are varied and sometimes debated, though it is generally accepted that electron transfer plays a critical role prior to the emission of light [38]. In this context, chemiluminescence can be seen as the inverse of the electron transfer process triggered by photoexcitation, where light is the end product rather than the initiator. When the energy level of the electron transfer state exceeds that of the excited state, photo-induced electron transfer is not feasible. However, if radical cations and anions (A^•+^ + B^•−^) are generated through chemical reactions, the recombination of these charges can lead to the formation of an excited state (C*). If this excited state (C*) is emissive, it results in the emission of light, defined as chemiluminescence, represented by Reaction (1):

A^•+^ + B^•−^ → C* + D → C + D + *hν* (1)

This process converts chemical energy into light energy, analogous to the reverse of photochemical electron transfer [39].

The chemiluminescence process involves three key steps: (1) formation of high-energy intermediates such as 1,2-dioxetane, 1,2-dioxetanone, and 1,2-dioxetanedione [40], which release sufficient energy to produce visible light (ranging from 40–70 kcal/mol); (2) decomposition of these high-energy intermediates and chemiexcitation to an excited state; and (3) emission of light from the excited state [39].

The quantum yield of chemiluminescence is an essential parameter for assessing the efficiency of different chemiluminescent systems. It is defined as the ratio of moles of photons emitted to moles of the limiting reagent, with the unit being einsteins per mole. For example, the chemiluminescence quantum yield of the luminol system is 1.29 × 10^−2^ einsteins/mol, indicating that 1 mol of luminol produces 1.29 × 10^−2^ mol of photons. The theoretical maximum quantum yield is 1 einstein/mol, meaning that 1 mol of the substrate can produce a maximum of 1 mol of photons. It is crucial to distinguish between chemiluminescence intensity and the number of photons (in counts), as intensity is influenced not only by the number of photons but also by their frequency [41,42].

Unlike photoluminescence, chemiluminescence does not require an excitation light source and accompanying excitation/emission filters. Thus, chemiluminescent optical biosensors produce a signal that can be measured directly using a photodetector, resulting in a simpler, compact device. The lack of an excitation source also improves signal-to-noise ratio with reduced background noise [37]. CL biosensors also demonstrate a wide dynamic range, allowing for the simultaneous detection of strong and weak luminescence [43].

To enhance the light emission of CL optical biosensors, advances in CL amplification have been essential, particularly the introduction of nanomaterials. A class of material that has garnered significant attention for amplification purposes is metallic nanoparticles (MNPs). Amplification has been achieved through a variety of MNP properties, notably MNP catalytic activity for higher reaction yields and surface plasmon resonance, resulting in metal-enhanced chemiluminescence (MEC) [44,45] improving the overall quantum yield. Likewise, the CL biosensor presented by Xu et al. exploited the catalytic capacity of copper nanoclusters as a nano-mimic enzyme for peroxidase [44].

A promising avenue for chemiluminescence optical biosensing is utilizing bioluminescence (Figure 1b), in which high-energy intermediates are synthesized via metabolic processes. Bioluminescence optical biosensors are particularly attractive due to the high efficiency of bioluminescence with the metabolism of luciferin, specifically exhibiting very high quantum yield [46]. Luciferin-based bioluminescence biosensors have been demonstrated to effectively detect the concentration of ATP with a linear relationship between analyte concentration and bioluminescence intensity [13,47,48].

### 2.2. Electrochemiluminescence (ECL)

Electrochemiluminescence (ECL) (Box 1) is a process that combines electrochemical reactions with chemiluminescence to produce light. In ECL, reactive species are generated at the surface of an electrode through electrochemical reactions involving the oxidation or reduction in a luminescent molecule (luminophore) and, in some cases, a co-reactant. The mechanisms underlying ECL can be categorized into three main pathways: the annihilation pathway, the co-reactant pathway, and the cathodic pathway [49,50].

The annihilation pathway involves the generation of both oxidized and reduced forms of the luminophore at the electrodes. These forms then interact (annihilate) to produce an excited state that emits light. This mechanism typically involves alternating the potential at the electrode to sequentially generate the radical cation and radical anion of the luminophore. For example, the luminophore [Ru(bpy)_3_]^2+^ is oxidized to [Ru(bpy)_3_]^3+^ and reduced to [Ru(bpy)_3_]^+^. When these species annihilate, they form the excited state [Ru(bpy)_3_]^2+^*, which then emits light as it returns to the ground state [43,49,50].

The co-reactant pathway utilizes an additional molecule (co-reactant) that participates in the electrochemical reactions to produce the excited state. The co-reactant is typically oxidized or reduced to form a reactive intermediate, which then reacts with the oxidized or reduced luminophore to generate the excited state. For instance, in the [Ru(bpy)_3_]^2+^/TPrA system, [Ru(bpy)_3_]^2+^ is oxidized to [Ru(bpy)_3_]^3+^, and TPrA is oxidized and then converts to TPrA^•^. The interaction between [Ru(bpy)_3_]^3+^ and TPrA^•^ forms the excited state [Ru(bpy)_3_]^2+^*, which emits light upon returning to the ground state [49]. Due to the direct relationship between the intensity of the emitted light and the concentration of the emitter or co-reactant, this form of ECL has been extensively utilized in analytical applications, especially in highly sensitive bioanalysis [51].

The cathodic pathway involves reduction reactions occurring at the cathode to generate the excited state [52]. In this pathway, the luminophore and/or co-reactant are reduced at the cathode to form reactive intermediates that produce the excited state. Although less common than the oxidative (annihilation and co-reactant) pathways, the cathodic path can be significant in certain systems. For example, [Ru(bpy)_3_]^2+^ can be reduced to [Ru(bpy)_3_]^+^, which then reacts with a reduced co-reactant to form the excited state [Ru(bpy)_3_]^2+^* that emits light as it returns to the ground state [49,53].

Box 1 **Electrochemiluminescence mechanisms and application of common co-reactants.**

**Electrochemiluminescence (ECL)** involves the generation of reactive species at the electrode surface through electrochemical reactions, including the oxidation or reduction in a luminophore and sometimes a co-reactant. ECL mechanisms can be categorized into three pathways: annihilation, co-reactant, and cathodic. The **annihilation pathway** involves generating both oxidized and reduced forms of the luminophore, which then interact to form an excited state that emits light. The **cathodic pathway** involves reduction reactions at the cathode, generating reactive intermediates that produce an excited state. The **co-reactant pathway**, which is particularly significant for its sensitivity in analytical bioanalysis, utilizes an additional molecule (co-reactant) that participates in the electrochemical reactions. In the [Ru(bpy)_3_]^2+^/TPrA system, [Ru(bpy)_3_]^2+^ is oxidized to [Ru(bpy)_3_]^3+^, and TPrA is oxidized to TPrA•. The interaction between [Ru(bpy)_3_]^3+^ and TPrA• forms the excited state [Ru(bpy)_3_]^2+^*, which emits light as it returns to the ground state. This mechanism’s direct correlation between light emission intensity and the concentration of the emitter or co-reactant makes it widely used in sensitive bioanalytical applications.

The most common ECL co-reactant systems include the following:

***1.***  ***[Ru(bpy)_3_]^2+^/TPrA:***
  - **Luminophore:** Tris(2,2′-bipyridyl)ruthenium(II) ([Ru(bpy)_3_]^2+^);
  - **Co-reactant:** Tripropylamine (TPrA).

This is one of the most extensively used systems due to its high efficiency and sensitivity in bioanalytical applications. The application of this co-reactant system that results in an emission of red ECL is as follows:

**Immunoassays**: The [Ru(bpy)_3_]^2+^/TPrA system is extensively used in immunoassays, where ECL tags help detect and quantify antigens or antibodies in samples [54,55,56,57,58,59,60,61,62]. This is crucial for disease diagnosis and monitoring. As an alternative labeling strategy, co-reactants employed in ECL reactions can be utilized for tagging biomolecules [63,64].
**DNA Analysis**: [Ru(bpy)_3_]^2+^-based DNA detection methods are instrumental in diverse areas such as clinical diagnostics, pathogen identification for infectious diseases, forensic analysis, and the diagnosis of genetically linked human diseases [55,65,66,67,68,69,70,71,72,73,74,75,76,77].
**Aptamer-based Biosensing**: The unique properties of aptamers, which are functional nucleic acids capable of specific target recognition, have been combined with ECL detection for the development of a multitude of aptasensors [78,79,80,81,82,83,84,85,86,87,88,89,90,91,92,93,94].

***2.***  ***Luminol/H_2_O_2_:***
  - **Luminophore:** Luminol (3-aminophthalhydrazide);
  - **Co-reactant:** Hydrogen Peroxide (H_2_O_2_).

Luminol is oxidized by hydrogen peroxide in the presence of a catalyst (often a metal ion like Fe^2+^ or a peroxidase enzyme) to form an excited state intermediate (3-aminophthalate). This intermediate then emits light as it returns to the ground state. The Luminol/H_2_O_2_ system is renowned for its applications in chemiluminescence (CL) and electrochemiluminescence (ECL) due to its strong blue light-emitting capabilities and reagent availabilities [95]. It is widely utilized in the following:

**Biological substrates**: Since H_2_O_2_ is the co-reactant of luminol, any substrate that produces H_2_O_2_ through enzymatic oxidation can be detected using this ECL system. Examples of these analytes include glucose [96,97,98,99,100,101,102,103], uric acid [104,105,106], cholesterol [107,108,109,110], l-lactate, and creatinine.
**Genetic detection**: DNA detection for clinical research [111,112] and miRNA sensing for cancer diagnosis [113,114,115].
**Immunosensing**: In immunoassays and other diagnostic tests where high sensitivity is required [116,117,118,119,120,121,122,123,124].

***3.***  ***Other pairs:***

There are also other ECL luminophore–co-reactant pairs. For [Ru(bpy)_3_]^2+^ and [Ru(phen)_3_]^2+^ luminophores, apart from TPrA, some other co-reactants, including dibutylamine (DBAE) and triethylamine (TEA), have been explored. Green ECL can be achieved using various luminophore and co-reactant pairs, including Ir(ppy)_3_ (tris(2-phenylpyridine)iridium(III)) and TPrA [105,125,126].

The general ECL process thus includes the electrochemical generation of reactive intermediates, the formation of excited states through interactions between these intermediates, and the emission of light as these excited states relax to the ground state. This ability to control the generation of excited states electrochemically makes ECL a powerful technique for various applications, including bioassays, sensing, and imaging.

Beyond the mechanistic aspects of ECL reactions, it is crucial to examine the spectroscopic and electrochemical characteristics of the reaction partners. These properties influence the thermodynamics and the resulting kinetics of the reactions that produce the excited state, thereby determining the efficiency of the ECL process.

The efficiency of electrochemiluminescence (Φ_ECL_) is defined by the ratio of photons emitted to the total number of electron transfer events during ECL reactions [51]. Φ_ECL_ comprises two components: Φ_ex_, which indicates the yield of excited states, and Φ_PL_, which represents the probability of photon emission from these excited states (analogous to the photoluminescence quantum yield).

(2) $$\Phi_{ECL}=\Phi_{PL}\times \Phi_{ex}$$

Φ_ex_ is influenced by the kinetics and thermodynamics of the reaction pathways and various experimental factors, such as the stability of precursors and their likelihood of undergoing side reactions. Conversely, Φ_PL_ is an intrinsic photophysical property affected by the solvent and the susceptibility of the excited state to quenching interactions.

Energetically, for an excited state to form from a bimolecular reaction, the energy available from the reactants must exceed the energy of the excited state product. Electrochemical experiments can often assess the free energy of these reactions using the redox potentials of the reactants. For instance, in ruthenium-based ECL, the energy of the excited state product (E_em_) can be estimated from the maximum emission wavelength (λ_max_ = 620 nm) using the equation E = hc/λ_max_ [51]. Here, h is Planck’s constant (4.13 × 10^−15^ eVs), and c is the speed of light (3.00 × 10^8^ m/s), yielding an E_em_ value of 2.05 eV. Given that the free energy of ECL pathways is 2.61 eV for annihilation and 2.98 eV for co-reactant pathways, these reactions sufficiently energize the 2.05 eV excited state of the ruthenium complex.

A central question in radiative and reactive intermediate electron transfer reactions concerns the population of excited states rather than the thermodynamically favored ground state. Marcus’ theory offers a compelling explanation [127,128]. It posits that electron transfer occurs on a significantly faster timescale compared to nuclear rearrangements, solvent reorganization, and bond vibrations. Consequently, when these reactions release substantial energy on an ultrafast timescale (shorter than a typical vibrational period), the system does not efficiently dissipate this energy through vibrational relaxation. This, in turn, makes the pathway leading to an electronically excited product more favorable.

In conclusion, understanding ECL efficiency and mechanisms is essential for optimizing its analytical applications. By examining factors influencing Φ_ECL_, such as kinetics, thermodynamics, and experimental conditions, researchers can improve ECL system performance. Future studies should focus on these areas to enhance ECL’s utility in various fields.

#### 2.2.1. Electrode Configurations in ECL

In an ECL device, the electrode setup is a critical component that influences the efficiency, sensitivity, and overall performance of the ECL system. Various configurations, such as the three-electrode setup, bipolar electrode ECL, and single-electrode ECL, each offer unique advantages and applications. The three-electrode setup, comprising a working electrode, reference electrode, and counter electrode, is the most commonly used configuration, providing precise control over the electrochemical environment and facilitating accurate measurements. Bipolar electrode ECL, on the other hand, simplifies the experimental setup by eliminating the need for multiple connections, making it suitable for miniaturized and portable devices. Single-electrode ECL further reduces complexity and can be advantageous in specific applications where space and simplicity are paramount. Understanding the distinctions of these different electrode setups is essential for optimizing ECL systems for diverse analytical and diagnostic applications.

##### Conventional/Three-Electrode ECL

The three-electrode cell is the classic configuration for electrochemiluminescence (ECL). It consists of three electrodes: the working electrode, the counter electrode, and the reference electrode [129]. This configuration was developed to address the limitations of earlier two-electrode systems, which often struggled to maintain a stable potential necessary for accurately measuring resistance at the interface of the working electrode and the solution [130]. In the three-electrode system, the reference electrode serves as a stable reference point for measuring and regulating the working electrode’s potential without allowing any current flow [130]. Consequently, this setup enables precise determination of potential changes at the working electrode, unaffected by fluctuations at the counter electrode [130]. The potential sweeps in this setup are typically characterized by a gradual rise in current followed by a subsequent decline, representing a two-step process involving the mass transfer of electroactive materials toward the working electrode’s surface and subsequent electron transfer reactions [130].

Recent advancements in three-electrode ECL systems have been notable. For example, a method was developed to use emissions on the counter electrode to enhance multicolor and potential-resolved ECL systems [131]. The multicolor ECL from mixed luminophores, Ir(ppy)_3_ and [Ru(bpy)]_3_^2+^, at glassy carbon working and counter electrodes, was investigated, analyzing the simultaneous ECL processes [131]. Under these conditions, spatially resolved emissions at different electrodes were observed, suggesting the potential for more multiplexed detection systems [131].

Additionally, new materials have been incorporated into ECL to enhance detection sensitivity. For instance, a folding microfluidic paper-based analytical device (μ-PAD) was developed using carbonate/carboxymethyl chitosan (CaCO_3_/CMC) hybrid microspheres for signal amplification [132]. These microspheres, coated in silver nanoparticles and immobilized with single-stranded DNA (ssDNA) strands, significantly augmented sensitivity.

##### Bipolar ECL

**Figure 2:**
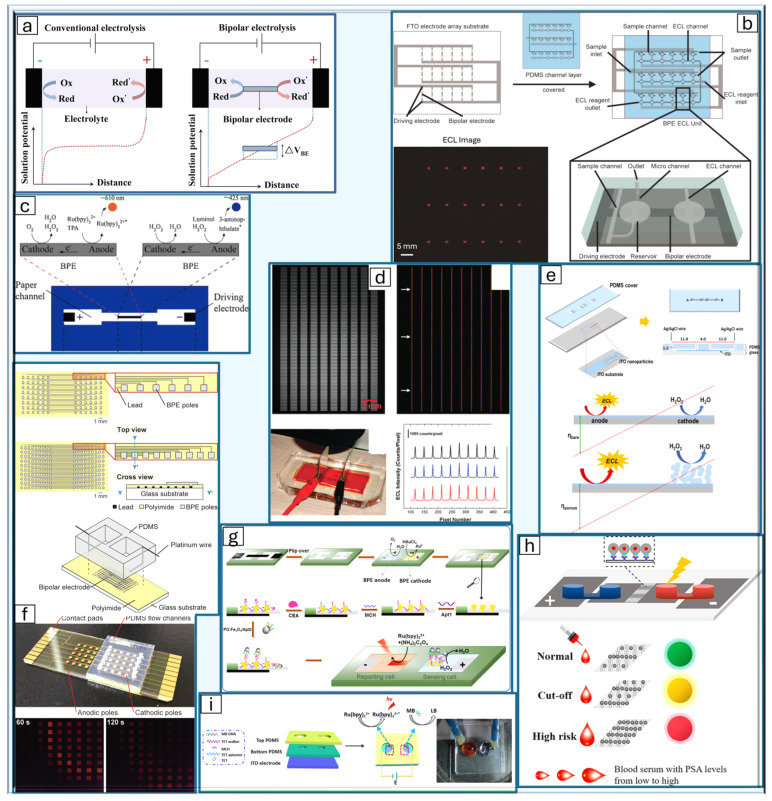
Electrochemiluminescence with bipolar configuration: (**a**) Electrode configuration in conventional ECL with a three-electrode setup (**left**), and in bipolar ECL (BPE) where a conductor is placed between the electrochemical cell, and the potential difference across the conductor drives the ECL reaction (**right**). Adapted with permission from [135], Copyright (2024). (**b**) Bipolar electrode array for multiplexed detection of prostate cancer biomarkers. Adapted with permission from [136], Copyright (2022). Liquid flow is directed by using a specialized channel structure that leverages differential flow resistance (**c**) paper-based BPE ECL for imaging and sensing. Adapted with permission from [137], Copyright (2024). Microfluidic channels are fabricated on filter paper using wax screen-printing, while carbon ink-based BPE and driving electrodes are also screen-printed onto the paper. (**d**) ECL using 1000 BPE arrays. Optical image (**top left**), ECL image (**top right**), electrochemical cell setup (**bottom left**), and ECL intensity profile (**bottom right**) of these arrays. Adapted with permission from [138], Copyright (2010). The simplified device uses a single pair of driving electrodes to activate a 500 μm × 50 μm electrode array, and the BPEs are placed in a shallow electrolyte pool between parallel plates to ensure uniform electric field (**e**) porous BPE using ITO nanoparticles. Top view and cross-section (**top**) and H_2_O_2_ sensing (**bottom**). Adapted with permission from [139], Copyright (2024). PDMS cover with 90 μm microchannels seals the BPE channel, separating detection and reporting channels. A nanoporous ITO layer detects H_2_O_2_, while a bare ITO layer reports ECL. The PDMS cover and ITO-coated glass form the complete BPE microchip. (**f**) BPE arrays for ECL imaging and multiplex sensing. Schematic of the device (**top**), real device, and ECL images (**bottom**). Adapted with permission from [140], Copyright (2022). The device features integrated cathodic and anodic poles and leads, insulated by a polyimide layer. Platinum and gold BPEs were fabricated using arrays of these poles. (**g**) Fabrication process of paper-based PBE aptasensor for detection of carcinoembryonic antigen. Adapted with permission from [141], Copyright (2024). Janus-like gold-coated Fe_3_O_4_ nanospheres were synthesized in this device and showed catalytic activity for H_2_O_2_ reduction. (**h**) Multicolor ECL using BPE configuration to detect prostate-specific antigen. Adapted with permission from [142], Copyright (2024). Selective ECL excitation is enabled by tuning the interfacial potential of the BPE poles in this device. (**i**) BPE ECL sensor using gold and glassy carbon beads for detection of tetracycline. Adapted with permission from [143], Copyright (2017). Au particles were selectively deposited on one side of a GCB electrode, controlling coverage from 0 to 45.3% using bipolar electroplating. This modified GCB allows for varying biomolecule conjugation.

Bipolar electroluminescence (BP-ECL) significantly differs from the conventional three-electrode setup, as the working electrode is replaced by the control of the solution potential [133]. A bipolar electrode (BPE) is a floating conductor that does not require a direct electrical connection to a power source or external circuit (Figure 2a). Instead, polarization is achieved by an electric field generated by two driving electrodes placed at opposite ends of an electrochemical cell. When a sufficiently high voltage is applied to the solution, simultaneous oxidation and reduction reactions occur at either end of the bipolar electrode [134]. This phenomenon occurs because the same number of electrons released by an oxidation reaction also participate in the co-occurring reduction reaction. This setup provides key advantages over the classic three-electrode system. As the BPE system is wireless and does not require an external power supply or a multichannel potentiostat, it is capable of conducting many simultaneous reactions with a single pair of driving electrodes and a power supply, making it useful for multiplexing and parallel sensing (Figure 2f) [133].

The difference between a closed and open bipolar system lies in the presence of a wall between the anodic and cathodic solutions. In a closed BP-ECL system, the cathode and anode solutions are physically separated so that the only path between the half cells is through the BPE (Figure 2i) [137].

The driving voltage *E* can be described by the following equation using Kirchhoff’s second law:

(3) $$E=∆\varphi \left(D2\right)-∆\varphi \left(C\right)+∆\varphi \left(A\right)-∆\varphi \left(D1\right)$$

where Δφ(D1), Δφ(C), Δφ(A), and Δφ(D2) are the potentials of driving electrode 1, the cathodic pole, the anodic pole, and driving electrode 2 with respect to the solution [140]. The driving voltage of an open bipolar electrode cannot be defined in this way, as it lacks the wall separating the two solutions [140].

To find the potential of the BPE, two parameters are considered: the overpotential along the electrode interface and the kinetic characteristics of the redox couple [138]. The potential difference between the solution and the two poles of the BPE, $∆E_{elec}$, represents the driving force available to couple the two reactions occurring simultaneously at either end of the BPE (Figure 2e). For the electrode to maintain its electroneutrality, these co-occurring reactions must have equal electron consumption and production [144]. $∆E_{elec}$ can be approximated by Equation (4) [138]:

(4) $$∆E_{elec}=\frac{E_{tot}}{l_{channel}}l_{elec}$$

where $E_{tot}$ is the voltage applied to the driving electrodes, $l_{elec}$ is the length of the electrode, and $l_{channel}$ is the distance between the driving electrodes.

Having gained an understanding of the principles and significance of bipolar electrochemiluminescence, a comprehensive review of the key research papers that have explored its diverse applications will now be conducted. Compared to the traditional three-electrode system ECL, BP-ECL has a much greater capacity for high-throughput detection (Figure 2d). To exploit this high-throughput capability of BP-ECL, a microfluidic array chip was developed to allow for multiplexed detection of prostate cancer biomarkers (Figure 2b) [136]. Immunosensing requires multiple steps, such as the loading of reagents and washing of the detection region, which limit its multiplexing capabilities [136]. To overcome this limitation, the design of the chip allows for the careful control of liquid flow through the channels, keeping the samples uniformly separated. These samples are then directed toward the BPE array, where they are captured by their corresponding antibodies. Glucose oxidase labeling allows for the production of hydrogen peroxide, which generates the ECL reaction [136].

##### Single-Electrode ECL

While bipolar electrode arrays have shown promise in areas of multiplexing, a significant drawback remains in the requirement for electrode arrays for these multiplex experiments. These electrode arrays are expensive and time-consuming to manufacture, which underscores the relevance of single-electrode ECL [133]. The basic premise behind the design of the single-electrode electrochemical system (SEES) is the reduction in the electrochemical cell to a single electrode. This is achieved by creating a potential difference along the surface of the electrode [106]. The initial SEES electrode was developed by applying an external voltage to both ends of the electrode, creating a potential gradient due to the varying resistance of the electrode [106]. The electrical potential produced by this process is directly proportional to the length of the cell and the applied voltage [104]. This method can then be utilized for ECL by generating excited states of luminol along the electrode, producing the ECL signal.

**Figure 3:**
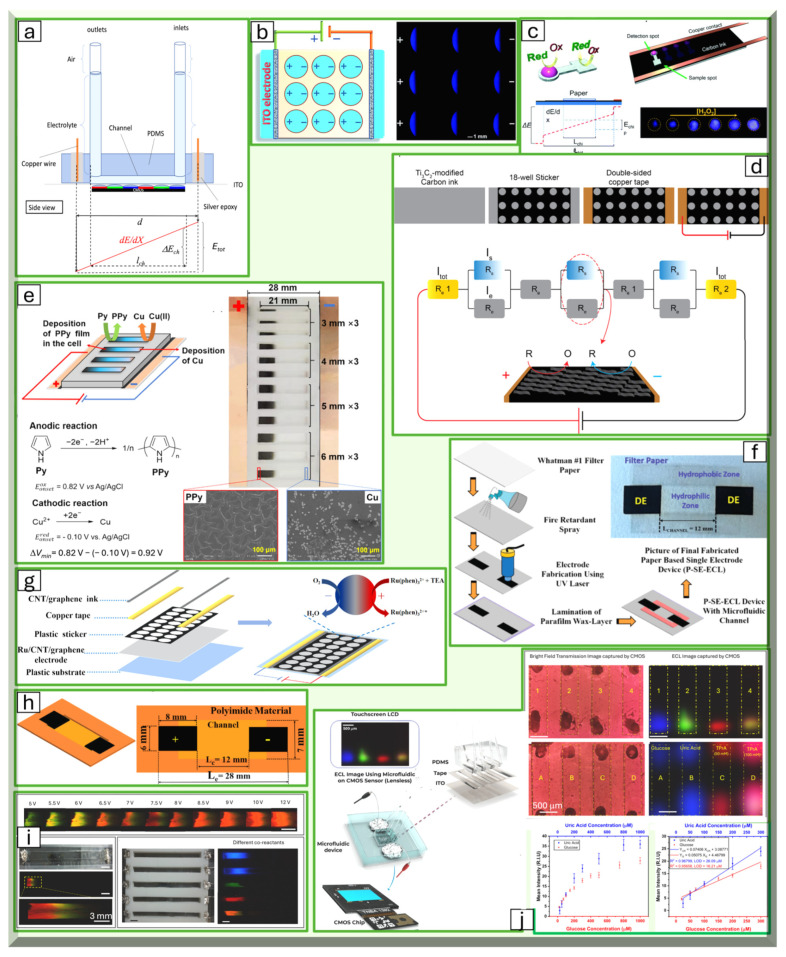
Electrochemiluminescence with single-electrode (SE) configuration: (**a**) schematic of SE ECL on CMOS image sensor (**top**), structure of SE configuration, and distribution of potential along the single electrode (**bottom**). Adapted with permission from [104], Copyright (2024). (**b**) First demonstration of SE-ECL, multiplex ECL using SE configuration. Adapted with permission from [106], Copyright (2018). SE-ECL for single and multiplex experiments are created by attaching labels with varying hole patterns onto ITO electrodes. (**c**) Paper-based SE-ECL on carbon ink. Adapted with permission from [146], Copyright (2022). A paper-based biosensor used glow sticks as a luminophore, detected by a smartphone. A two-compartment paper device enabled separate optimization of sensing and detection reactions. (**d**) Fabrication of multiple-well SE-ECL on carbon ink (**top**) and equivalent circuit (**bottom**) for immunoassay application. Adapted with permission from [147], Copyright (2023). This paper-based SE-ECL, featuring a perforated sticker on a carbon ink electrode, utilized antibody immobilization and Co–Pt nanoparticles for sensitive SARS-CoV-2 detection. (**e**) SE-ECL with multiple channels fabricated by punching polypyrrole film and attaching it to the carbon ink layer (**top**) and ECL reactions on the cathodic and anodic side of each well (**bottom**). Adapted with permission from [148], Copyright (2023). (**f**) Paper-based SE-ECL fabricated by reducing graphene oxide via laser and wax printing to form microfluidic channels for glucose and lactate sensing. Adapted with permission from [149], Copyright (2024). (**g**) Multiplex SE-ECL fabricated using Ru(phen)_3_^2+^ modified carbon nanotube/graphene film and a plastic sticker with 24 holes for dopamine detection. Adapted with permission from [150], Copyright (2024). (**h**) SE-ECL device fabricated using laser-induced graphene and polyimide film. Adapted with permission from [151], Copyright (2024). (**i**) First multicolor SE-ECL device fabricated by 3D-printed channels. Multicolor ECL using different potential in different channels (**top**), multicolor ECL using potential gradient along the SE (**bottom left**), photo of the 3D-printed SE-ECL (**bottom center**), and multicolor SE-ECL on different channels (**bottom right**). Adapted with permission from [105], Copyright (2024). (**j**) Miniaturized and microfluidic-integrated SE-ECL on CMOS image sensor: schematic of the device (**left**) and detection of multiple analytes including glucose and uric acid with this device (**right**). Adapted with permission from [105], Copyright (2024).

The potential difference between the ends of the cell ($∆E_{c}$) primarily depends on the ratio between the electrochemical cell’s length $L_{c}$ and the length of the plastic film [106]. When $\Delta E_{c}$ is sufficiently large, reduction and oxidation will occur simultaneously at either end of the microelectrochemical cell, resulting in the following formula (Figure 3a) [104,106]:

(5) $$\Delta E_{c}=\frac{E_{tot}}{L_{c}}\times L_{c}$$

The very first single-electrode ECL system was developed using an indium tin oxide (ITO) electrode and a self-adhesive label with holes poked into it [106]. This system was designed for multiplexed experiments targeted towards high-throughput analysis, as each hole serves as an independent microelectrochemical cell. Initially, this system demonstrated its use in detecting luminol, hydrogen peroxide, uric acid, and glucose (Figure 3b) [106]. Later, the single-electrode system was adapted to be wireless by incorporating wireless energy transfer modules and a rectifying diode [145]. The resulting system exhibited a linear range from 1 to 150 μM and a detection limit of 0.26 μM. Additionally, visual detection was carried out using a smartphone, indicating promising potential for point-of-care applications.

In 2021, a graphene-based single electrode on a polyimide substrate was developed for the detection of multiple analytes using a luminol-based chemical reaction (Figure 3h) [151]. The electrodes were fabricated with a CO_2_ infrared laser, forming laser-induced graphene on the surface of the polyimide substrate. This differs from the original single electrode developed on an ITO substrate, allowing for a miniaturized form with applications in numerous industries. The system was later adapted for the detection of vitamin B_12_ [152].

In 2022, several single-electrode systems using paper as the substrate were developed. One such system involved a disposable paper-based single electrode fabricated with carbon paint and commercial glow stick dye [146]. When an electric field is applied to the device, anodic and cathodic reactions occur at either end, separated by the dumbbell shape of the paper. The materials used were deliberately chosen to be common to maximize accessibility. Another paper-based single electrode was fabricated by embedding reduced graphene oxide through blue laser ablation, demonstrating effectiveness in the detection of glucose and lactate (Figure 3f) [149].

Building on the advancements in single-electrode ECL systems, the strong π-π stacking interaction of carbon nanotube (CNT)/graphene was exploited to immobilize [Ru(phen)]_3_^2+^ to create a modified single electrode (Figure 3g) [150]. The resistance of the [Ru(phen)]_3_^2+^ modified CNT/graphene film then induces SEES when a voltage is applied, allowing for the visual detection of ECL. This system has been applied for dopamine detection since dopamine quenches this ECL.

The potential applications for SEES in high-throughput systems have also been expanded by several researchers. A single-electrode ECL for a high-throughput immunoassay was developed by using a carbon ink screen electrode, with cardiac troponin I (cTnI) antibodies immobilized along its surface [153]. When these antibodies bind to cTnI, electron transfer along the electrode is inhibited, resulting in a decrease in ECL intensity. Additionally, a gradient potential distribution typically used in bipolar electrochemistry was adapted for use on a single electrode by modifying a classic ITO single-electrode setup through the electrodeposition of a gradient polypyrrole (PPy) conductive film, resulting in a high-throughput electroanalysis system (Figure 3e) [148].

Most recently, efforts to increase the sensitivity of single-electrode electrochemical biosensor systems have been made by modifying the surface of the electrode with MXene structures and Co-Pt nanoparticles (Figure 3d) [147]. MXene structures have garnered interest due to their properties, such as high electron conductivity and ion adsorption, which enable their use in sensitive ECL systems. Co-Pt nanoparticles were incorporated due to their effectiveness in enhancing the ECL of luminol in the presence of H_2_O_2_. Finally, by incorporating a Raspberry Pi computer and an artificial neural network, a system was created that significantly enhances the sensitivity of detection [133].

## 3. From Point Detectors to Optical Image Sensors

In emission-based optical biosensing, a photodetector is essential for detecting light and converting it into an electrical signal. Photodetectors operate based on the photoelectric effect, a phenomenon where incident photons with sufficient energy are absorbed by a material, causing the ejection of electrons known as photoelectrons. The photoelectric effect was first documented by Heinrich Hertz, with further experiments by Philipp Lenard and Robert Millikan demonstrating the dependence of the number of emitted photoelectrons on the intensity and wavelength of the incident light, respectively [154,155,156]. These findings were later explained by Albert Einstein [157]. According to Einstein’s explanation, the energy of incoming radiation is quantified as *hn*, where *n* is the frequency of light. Therefore, the energy imparted to a photoelectron is related to the wavelength of the absorbed light. Assuming that each photon with sufficient energy excites only one electron, the light intensity, or rate of photons, must correspond to the number of photoelectrons emitted.

Several important qualities are used to evaluate the performance of a photodetector, including sensitivity, noise, spectral response, response time, and quantum efficiency [158,159,160]. Sensitivity describes a photodetector’s ability to detect low levels of light. This is particularly crucial for chemiluminescence optical biosensors due to the weak emission typically produced by chemiluminescence. However, achieving high sensitivity can also introduce unwanted noise. Therefore, it is important to optimize the photodetector’s gain to achieve sufficient sensitivity while minimizing noise. The spectral response of a photodetector refers to the range of light frequencies it can detect. A broader spectral response allows for a wider range of applications. For real-time monitoring, the response time of a photodetector is critical, with shorter response times enabling more accurate temporal measurements. Lastly, quantum efficiency, defined as the ratio of photogenerated charge carriers to incident photons, indicates how efficiently a light signal is converted into an electrical signal. High quantum efficiency is essential for low-light and high-accuracy applications.

While photodetectors may follow the same fundamental photoelectric principle for light-to-electric signal conversion, the way this conversion is achieved can greatly affect the detector’s performance. In the following sections, two-point detectors (Figure 1c), photomultiplier tubes (PMTs) and avalanche photodiodes (APDs), will be introduced and compared to imaging-based detectors (Figure 1d), including charge-coupled devices (CCDs) and complementary metal–oxide–semiconductor (CMOS) sensors.

### 3.1. Photomultiplier Tubes (PMTs)

The initial development of PMTs dates back to the early twentieth century, coinciding with the development of vacuum tubes and advancements in the understanding of photoelectric phenomena. In the late 1800s, the discovery of the photoelectric effect by Heinrich Hertz and its further explanation by Albert Einstein demonstrated the ejection of electrons from a material when exposed to light [154,157]. However, the first applications of this phenomenon were not realized until the invention of the phototube in the 1920s. Phototubes comprised a photosensitive material that ejected electrons upon exposure to light, producing a small and often too weak electric current, thus necessitating the need for amplification methods.

The innovation of the PMT soon followed, with Leonid Kubetsky’s work on secondary emission for electron amplification eventually leading to the development of the first commercially available PMTs by the 1930s [161]. After World War II, PMT technology saw rapid improvements driven by the demands of research and industry. Advancements in vacuum technology, photosensitive materials, and amplification techniques greatly enhanced PMT performance and reliability. The modern PMT is a highly sensitive instrument capable of single-photon detection with high efficiency and low noise across a wide spectral response. Recent innovations in the manufacturing processes of PMTs have resulted in more robust and versatile devices, making them suitable for a variety of applications, including medical diagnostics and astronomy [162,163].

PMTs offer several key advantages, including high sensitivity, fast response time, high gain with low noise, and a wide spectral response. With the development of high quantum efficiency photocathodes, dynodes, and optimized electronics, PMTs are capable of sensing single-photon emissions by applying high amounts of gain [164]. Due to the controlled manner of amplification, with each dynode adding predictable amounts of gain, PMTs can achieve high gain with low over-multiplication noise while exhibiting a linear response over a wide range of incident light intensities [165]. Additionally, since amplification does not occur at the photocathode, PMTs benefit from a large variety of available materials for photocathodes, enabling a wide spectral response.

While PMTs are an excellent choice for high-sensitivity, low-noise applications, they also have several drawbacks. Since PMTs are made from glass vacuum tubes, they are generally quite delicate. Additionally, PMTs are sensitive to magnetic fields, which can interfere with the trajectory of emitted electrons passing through the vacuum tube, reducing efficiency, causing gain variations, and adding signal noise [164,166,167]. This problem can be addressed using magnetic shielding or active compensation coils [164,167]. For point-of-care (POC) applications, PMTs may not be suitable because they require a high voltage power supply for amplification [164]. Furthermore, the combination of specialized photocathode and dynode materials, complex design, magnetic interference countermeasures, and power supply requirements results in high costs. While solutions to these problems may be available, such as, for example, the implementation by Hamamatsu of voltage multipliers with programming voltage, applications of PMTs for POC applications are currently less ideal.

PMTs have been utilized in numerous experimental designs across various applications. For example, the chemiluminescence biosensor for detecting cholesterol by Xu et al. employed a PMT. Multiple fiber-optic-based biosensors for detecting sorbitol have also been demonstrated using PMT detectors [168,169]. In particular, Gessei et al. [169] devised and evaluated the performance of a biosensor using both a spectrometer and a PMT. The study found that using a PMT for detection improved the signal-to-noise ratio by approximately 2.6 times. This high sensitivity of the PMT was crucial for detecting low concentrations of sorbitol that were undetectable by the spectrometer.

### 3.2. Avalanche Photodiodes (APDs)

An avalanche photodiode is a semiconductor device used for converting light into an electrical current. The initial concept for the APD originated from the seminal work of Jun-ichi Nishizawa in the 1950s, who proposed the avalanche multiplication effect in semiconductors. In the following decade, research in semiconductor materials, particularly silicon and germanium, led to the development of more efficient photodiodes and early versions of APDs [166]. By the late 1980s and early 1990s, APDs had become commercially available thanks to significant advancements in semiconductor fabrication [166]. Since then, APDs have continued to undergo further development to improve sensitivity and speed, reduce noise, and increase bandwidth [170,171,172]. These properties have made APDs suitable for use in several industries and technologies, including telecommunications, light detection and ranging (LiDAR), and medical imaging [170,171,172].

Similar to PMTs, APDs offer high sensitivity and gain [171]. Additionally, APDs generally have high quantum efficiency due to their intrinsic design and material properties. Since APDs internally amplify current through impact ionization, charge carriers are contained and can efficiently interact with other atoms in the depletion region. This mechanism also results in fast response times, making APDs suitable for high-speed detection applications [171]. Furthermore, optimization of the depletion region and electric field reduces the probability of electron–hole recombination, leading to greater charge accumulation [173]. For optimal photoemission, semiconductor materials can be tailored for specific wavelengths of light with high absorption to promote charge carrier generation [171].

The internal gain mechanism of APDs, while providing many benefits, is also responsible for the amplification noise associated with APDs [171]. Unlike the predictable gain offered by PMTs, impact ionization in APDs is stochastic. Thus, while impact ionization amplifies the signal current, it also generates statistical noise known as Poisson noise. APDs are also limited in maximum gain. Since a high reverse-bias voltage must be applied to the p-n junction for impact ionization, APDs are limited by the breakdown voltage of the diode. This issue has been addressed to some degree in Geiger-mode single-photon APDs (SPADs). However, while APDs operate as linear amplifiers, SPADs operate using a very strong electric field to trigger self-sustaining impact ionization that is quenched to stop the avalanche process and give time to recover [174]. Thus, SPADs must operate at a lower count rate to produce single-photon detection events as signal pulses.

A whole-cell bioluminescent sensor for detecting *E. coli* presented by Daniel et al. used an SPAD for signal detection [175]. While PMTs were considered for this application, it was determined that PMTs were too large and unsuited for mobile biosensing applications and relatively expensive compared to SPADs. As a silicon-based photon counting element, SPADs could also be integrated with the signal processing unit, allowing for compact sensor integration. A similar bioluminescent sensor also used an SPAD for the detection of *E. coli* [176]. This study also considered PMTs as well as charge-coupled devices (CCDs) as alternative detectors but ultimately selected SPADs due to their high quantum efficiency, relatively low cost, and low operating voltage. The sensor was integrated into a small lightproof container to create a compact bioluminescent biosensor. Performance testing of the sensor demonstrated a proportional increase in SPAD readout with the concentration of generated oxyluciferin, indicating an increase in *E. coli* activity.

### 3.3. Plate Readers

For measuring individual samples, PMTs and APDs provide a sensitive, high-gain, fast, and low-noise tool for light measurement. However, as point detectors, scaling to larger sample sizes proves to be challenging, with low throughput and a labor-intensive and time-consuming process. To resolve this, the multiplexed integration of PMTs into plate readers can significantly improve the viability of PMTs in higher-throughput applications.

The first plate readers appeared in the 1970s as simple devices designed primarily to measure absorbance for colorimetric assays [177,178]. Future innovations soon incorporated additional detection modes, including fluorescence and luminescence assays [179,180]. Fast-forwarding to the modern era, current plate reading devices feature several improvements, including automation, user-friendly software integration, and multi-unit detectors, to meet the demands of drug discovery, clinical diagnostics, and life science use cases [180,181].

Plate readers are widely used in laboratories for their efficiency and versatility in high-throughput screening, enzyme-linked immunosorbent assays (ELISAs), and other biochemical assays. One of the primary advantages of plate readers is their ability to rapidly process multiple samples simultaneously, significantly increasing productivity and data throughput [182]. They offer high sensitivity and accuracy in detecting absorbance, fluorescence, and luminescence, which are essential for quantitative analyses [183]. Additionally, their automation capabilities reduce human error and variability, enhancing the reproducibility and reliability of results [184]. However, plate readers also have drawbacks. The initial cost of purchasing a plate reader can be substantial, and the maintenance and calibration required to ensure accurate readings can be time-consuming and expensive [185]. Furthermore, most old plate readers may not be suitable for all types of assays, particularly those requiring real-time kinetic measurements, where more specialized equipment might be needed [181]. Thus, plate readers are generally ill-suited for POC and PON applications.

### 3.4. Optical Image Sensors

Image sensors consist of an array of point photodetectors that form pixels, the smallest spatially resolvable units with submicron size in state-of-the-art technologies, enabling spatial resolution. The structure of image sensors involves an array of microlens at the top, followed by a color filter, photodiodes, and electronic components for converting the collected photons into electrical currents [186,187]. The layer of microlenses at the top of the pixel array focuses incoming light onto the photodiodes, enhancing the fill factor determined by the ratio of a pixel’s photosensitive area to its total area. The mosaic color filter array commonly adopts a Bayer filter arrangement with a 1:2:1 ratio of red (R), green (G), and blue (B) to separate the incoming light into RGB color channels, enabling color resolution. Since its invention in 1980, the pinned photodiode (PPD) has been widely adopted in most image sensors [188]. In a PPD, a thin p-type layer located on top of the n-type region of the photodiode stabilizes the surface potential of the photodiode, resulting in lower noise, higher quantum efficiency, and lower dark current [188]. Electronic components transform incoming photons into voltage signals, with amplifiers to increase the signal-to-noise ratio and enhance dynamic range, while analog-to-digital converters (ADCs) transform the voltage signals into digital values, and digital I/O and column decoders store and output the digitized results [189].

The two main types of image sensors are charge-coupled devices (CCDs) and complementary metal–oxide–semiconductor (CMOS) image sensors. CCDs and CMOS image sensors differ fundamentally in their architecture and signal readout mechanisms, with CCDs built on p-doped metal–oxide–semiconductor (MOS) capacitors and CMOS image sensors using p and n-doped MOS field-effect transistor (MOSFET).

#### 3.4.1. Charge-Coupled Devices (CCDs)

The CCD image sensor was the first solid-state image sensor developed. After CCDs were invented in 1969 at Bell Labs by Boyle and Smith [190], the first CCD image sensor was reported in 1970 [191]. A CCD consists of an array of connected capacitors that transfer electric charge to neighboring capacitors. The electric charge is generated when photons hit the photoactive area of the capacitor array and subsequently transferred across the series of capacitors [192]. A common readout point with a charge amplifier is located after the last capacitor to convert the charge into a readable voltage [192]. The use of a common readout point outside of the pixels and reduced circuitry in each pixel of CCD contributes to a high fill factor, leading to better photon collection efficiency that creates higher image quality with lower noise. However, CCDs suffer from slower readout speed and consume more power due to the continuous sequential charge transfer process. Since CCDs generally provide superior image quality and better performance in low-light conditions, they are ideal for applications such as astronomy, professional photography, and medical imaging [193].

#### 3.4.2. Complementary Metal–Oxide–Semiconductor (CMOS) Sensors

CCDs require high charge transfer performance, leading to several challenges, including manufacturing difficulties and issues with operating at low temperatures and high frame rates [194]. An active pixel sensor (APS), where each pixel is equipped with a transistor, can overcome these inherent problems with CCDs [194]. The first CMOS image sensor using APS was invented in the mid-1990s by Fossum et al. at NASA Jet Propulsion Laboratory [195]. PMOS and NMOS transistors exhibit low electrical resistance between their source and drain when a low and high voltage is applied, respectively. Therefore, connecting the sources and drains of PMOS and NMOS transistors in CMOS transistors results in reduced electrical resistance across a broader applied voltage range, leading to lower power consumption and less heat generation, which contribute to reduced noise [196].

CMOS image sensors were initially front-side illuminated (FSI), where the photodiodes are located below the electronic interconnects. Newer generations of CMOS image sensors have adopted back-side illumination (BSI) by placing the photodiodes above the interconnects to enhance the photon collection efficiency [197]. The state-of-the-art technology in the new generation of CMOS image sensors includes notable advancements such as PureCel^®^Plus Technology. This technology advances CMOS image sensors through several key innovations, including the implementation of a buried color filter array (BCFA) and deep trench isolation (DTI) [198]. The BCFA significantly enhances the ability to capture light from various incident angles. Meanwhile, DTI minimizes crosstalk by creating isolation walls between pixels within the silicon, thereby improving chief ray angle (CRA) tolerance. The second generation of PureCel^®^Plus further refines DTI for superior pixel isolation and enhanced low-light performance. Additionally, composite metal grid (CMG) technology boosts pixel sensitivity by forming walls above the silicon surface, which further reduces pixel color crosstalk. These advancements collectively contribute to superior image quality, making PureCel^®^Plus Technology a leading innovation in CMOS image sensor technology.

Unlike CCDs, CMOS image sensors have individual readout circuits on each pixel, enabling faster, parallel readout of image data with lower power consumption as well as random access to pixel information [197]. Although the pixel-level readout circuitry in CMOS image sensors reduces the area available for photon capture compared to CCDs, resulting in historically lower signal-to-noise ratio, advancements such as BSI have significantly improved their performance, offering comparable image quality. Since CMOS image sensors are more power-efficient, they are suitable for portable devices that are battery-powered, such as smartphones [199] and cameras. CMOS image sensors also offer faster readout speeds and greater flexibility in data handling, making them ideal for high-speed imaging and applications requiring rapid real-time processing. These characteristics make CMOS image sensors ideal optical readers for POC biosensors where portability and fast readout are essential.

### 3.5. Parameters That Affect the Analytical Performance of a Biosensor

Biosensors are analytical devices that exploit biological or chemical reactions to generate measurable signals proportional to the concentration of a target analyte within a sample. The analytical performance of biosensors is influenced by various parameters, which can be categorized into two main groups: experimental parameters and component parameters [200]. Experimental parameters include factors such as applied potential, scan rate, frequency range, pH, temperature, incubation time, analyte concentration, and mixing speed. Each of these parameters plays a crucial role in determining the sensitivity, selectivity, and overall efficiency of the biosensor [200]. For instance, the applied potential can affect the electron transfer processes, while the scan rate influences the kinetics of the electrochemical reactions. The frequency range is pertinent in impedance-based biosensors, as it determines the response characteristics. Additionally, the pH and temperature conditions are critical, as they can alter the biochemical activity and stability of the biological recognition elements. Incubation time impacts the interaction between the analyte and the bioreceptor, and analyte concentration is directly related to the biosensor’s detection limit and dynamic range. Lastly, the mixing speed ensures homogeneity in the sample solution, thereby affecting the reproducibility and accuracy of the measurements.

The analytical performance of a biosensor is also intricately linked to several key component parameters. A typical biosensor system comprises three key components: biorecognition elements, transducers, and output systems. The selection of the transducer, which converts a physicochemical change into a measurable signal, is crucial. Common choices include electrochemical, optical, piezoelectric, and thermal devices, each offering specific signal conversion advantages [201]. Equally important is the selection of the biorecognition element, the biomolecule responsible for target recognition. Enzymes, antibodies, nucleic acids, and others can be employed depending on the specific analyte. The interaction between the target and the biorecognition element triggers a physicochemical change that the transducer can detect [202].

To ensure efficient target-biorecognition element interaction, a suitable immobilization technique must be chosen. Techniques such as adsorption, entrapment, encapsulation, covalent attachment, and cross-linking significantly influence the efficiency of immobilization and, consequently, the overall performance of the biosensor [203]. Furthermore, the selection of an appropriate support material is essential. Ideal support materials should possess excellent chemical and mechanical stability, offer reactive functional groups for biomolecule attachment, provide a high surface area, and exhibit inert, biocompatible, and cost-effective characteristics [204]. These materials can be broadly categorized as organic (e.g., alginate and chitosan) or inorganic (e.g., mesoporous silica and nanoparticles) [205]. Finally, additional constructional parameters include the selection and ratio of other biosensing components like crosslinkers, surfactants, and signal enhancers. The chosen coating method on the electrode surface (e.g., electrodeposition and drop-casting) also significantly affects the biosensor’s performance [206,207]. By carefully considering these constructional parameters, researchers can optimize biosensor design for superior analytical performance.

A biosensor’s effectiveness hinges on its ability to accurately detect and quantify an analyte within a specific concentration range. This performance is governed by a complex interplay of factors affecting the linear range, sensitivity (represented by the slope of the linear range), and limit of detection (LOD).

*Linear Range:* The linear range, where the response correlates directly with analyte concentration, is influenced by bioreceptor properties [208]. High-affinity bioreceptors can lead to a narrower range due to rapid saturation at low concentrations. Conversely, lower affinity interactions might extend the range but compromise sensitivity [209,210,211]. The transducer’s characteristics, particularly its sensitivity and dynamic range, also play a role. Highly sensitive transducers might exhibit a limited linear range. Surface coverage of bioreceptors can further impact this range, with higher densities causing faster saturation. Mass transport limitations, affecting analyte arrival at the sensor surface, can cause deviations from linearity at higher concentrations. Fortunately, strategies exist to manipulate the linear range. Signal amplification mechanisms can extend it by enhancing the sensor response at low concentrations. Additionally, sensor design considerations, such as the geometry and size of the sensing element, can improve mass transport, leading to a wider linear range. Environmental factors like temperature, pH, ionic strength, and sample matrix interferences can also affect linearity. Optimizing these parameters is crucial for reliable measurements.

*Sensitivity (slope of the linear range):* The slope of the linear range, representing the sensitivity of the biosensor, is influenced by several factors. The affinity of the bioreceptor for the analyte directly impacts sensitivity, with high-affinity interactions producing a steeper slope. The efficiency of the transducer in converting biorecognition events into measurable signals is crucial; more efficient transducers generate larger signal changes per unit concentration of analyte, resulting in a steeper slope [212,213]. Bioreceptor density on the sensor surface affects sensitivity, as higher densities enhance the likelihood of analyte binding. Signal amplification strategies, such as enzymatic reactions or nanoparticle use, increase sensor response, steepening the slope. The surface chemistry and immobilization methods employed can preserve bioreceptor functionality, optimizing sensitivity. Mass transport effects, ensuring efficient analyte delivery to the sensor surface and minimizing diffusion limitations, also contribute to a steeper slope. Optimal environmental conditions and reduced interference from non-specific binding maintain a high sensitivity, while sensor design factors such as electrode material and surface area further influence the slope.

*Limit of Detection***:** The limit of detection (LOD) of a biosensor, indicating the lowest detectable analyte concentration, is determined by various factors. The signal-to-noise ratio (SNR) is critical, as a higher SNR allows better distinction of the analyte signal from background noise, lowering the LOD. Bioreceptor affinity and specificity enhance the detection of low analyte concentrations, improving the LOD. The transducer’s intrinsic sensitivity also affects the LOD, with more sensitive transducers detecting smaller signal changes. [214,215]. Effective surface chemistry and immobilization techniques that maintain bioreceptor activity enhance the LOD. Signal amplification methods increase the generated signal per analyte molecule, lowering the LOD. Sample volume and pre-concentration techniques can improve the LOD by increasing the analyte molecules available for detection. Efficient mass transport to the sensor surface ensures that low analyte concentrations reach the bioreceptor sites, enhancing detection capabilities. Minimizing background interference and optimizing environmental conditions such as pH and temperature improve the bioreceptor–analyte interaction, lowering the LOD. Advanced data processing and analysis techniques further distinguish the signal from noise and compensate for background interference, improving the LOD.

*The performance of optical biosensors*, in particular, directly depends on the performance of the optical detector. As shown in Table 1, different types of optical detectors, such as PMT, APD, CCD, and CMOS sensors, exhibit distinct characteristics that affect their performance in biosensing applications. PMTs and APDs are highly sensitive with substantial gain, making them suitable for detecting low-intensity signals. CCDs and CMOS sensors, while less sensitive in comparison, offer sufficient sensitivity for most biosensing applications, with CMOS sensors improving rapidly in this aspect [216].

PMTs generally have low noise but can be affected by dark currents and afterpulsing. APDs exhibit higher noise levels, particularly in high-gain settings. CCDs are known for their low noise, particularly when cooled, whereas CMOS sensors have historically had higher noise but are continually improving with technological advancements [218]. CCDs excel in providing a high dynamic range and full well capacity, allowing for the detection of both low and high signal intensities without saturation. CMOS sensors are catching up, offering a competitive dynamic range and full capacity in modern designs. PMTs and APDs, while highly sensitive, can have limited dynamic ranges in comparison.

By carefully optimizing these factors, the performance of biosensors can be significantly improved, allowing for more accurate and sensitive detection of target analytes across a range of applications.

## 4. Integration of Chemiluminescence, Optical Image Sensors, and Microfluidic Components

Any biosensing/bioimaging device requires a detector. For the system to be fully independent and stand-alone, this detector must be integrated with other components of the system.

Despite the excellent sensitivity of single-point detectors, achieved through electron multiplication or avalanche effects, their application at the point of care remains limited. Efforts have been made to integrate these detectors with optical fibers to extend their use beyond the laboratory [219], but challenges persist due to their high voltage requirements, size, and cost. As an alternative, integrating optical detectors with analytical sensors using cellphone cameras has been explored. As smartphones become increasingly powerful and abundant, the feasibility of adapting their features into point-of-care sensing applications grows, making smartphone-integrated CL biosensors an exciting opportunity for accessible, affordable, and effective health monitoring tools. Numerous studies have employed cellphone cameras as biosensor detectors [220,221,222,223,224,225,226,227,228,229,230,231,232,233,234,235,236,237,238,239,240,241,242,243,244,245,246,247,248]. However, the variability in camera quality, sensor types, and focal planes across different cellphone models necessitates additional calibration steps, hindering the widespread application of this approach. Additionally, in resource-poor areas, access to cellphones with high-quality cameras is often limited.

Traditional bioimaging relies on magnification via lenses to achieve high spatial resolution. Advancements in digital sensor technology have led to the development of lens-based digital imaging. This technique combines magnifying lenses with high-resolution digital sensors, enabling rapid image acquisition and facilitating storage and processing. Notably, the convergence of CMOS image sensors, commonly found in mobile phones, with external magnifying lenses paves the way for portable and user-friendly microscopic imaging [199,249,250]. Lens-based imaging presents inherent trade-offs. Firstly, achieving high spatial resolution often comes at the expense of a reduced field of view (FOV) [251]. FOV denotes the total observable area captured by the sensor [199]. Secondly, lenses can introduce optical aberrations, manifesting as defocus and image distortion [249]. Finally, lens-based systems typically generate images with only intensity contrast, limiting the extraction of three-dimensional (3D) information. In contrast, lensless imaging overcomes limitations inherent to lens-based systems. It achieves aberration-free high-resolution images while preserving a large FOV. In lensless imaging, spatial resolution is defined by the image sensor’s pixel size and signal-to-noise ratio (SNR). The FOV, on the other hand, is directly proportional to the active area of the image sensor, reaching up to 30 mm^2^ for CMOS and 20 mm^2^ for CCD sensors, with pixel sizes as small as 0.7 μm [196,252]. For comparison, a conventional bench-top optical microscope equipped with a 10× objective lens (numerical aperture of 0.2) offers a limited field of view (FOV) of less than 4 mm^2^ and a theoretical spatial resolution of 1.5 μm, significantly restricting the sample area captured in a single image [253]. Additionally, lensless digital holographic imaging provides the distinct advantage of acquiring depth-resolved 3D information. Moreover, the absence of lenses translates to enhanced portability and cost-efficiency for these systems.

Integrated lensless optical systems can be categorized into two main types based on the signal they detect from the sample: shadow-based systems and luminescence-based systems. Shadow-based systems record a signal representative of the sample’s physical shape [186], while luminescence-based systems detect photons emitted by the sample. For enhanced ease and flexibility in sample handling, these two optical platforms were additionally integrated with optofluidic techniques. Within optofluidics, microfluidic devices enable precise manipulation of samples directly above the sensor arrays.

### 4.1. Integrated CL-Based Optical Systems

Lensless luminescence imaging offers distinct advantages. This technique captures the luminescence emitted by the sample, leading to enhanced optical contrast. Additionally, it yields quantified results in the form of light intensity, enabling the calculation of analyte concentration [196].

CL-based lensless imaging offers a versatile approach to biomolecule quantification [254]. This technique relies on placing the sample directly on the image sensor surface and capturing light emitted during chemiluminescent reactions [255,256]. CL imaging allows for the detection of various biomolecules, ranging from toxins [255] to disease markers [256], making it valuable for applications in food safety [13], diagnostics, and health monitoring. BL-based lensless imaging utilizes genetically engineered yeast or bacteria cells [257]. These cells express recognition elements that activate luciferase enzymes upon analyte interaction, leading to bioluminescence emission. The bioluminescent cells are then immobilized on or transferred to the image sensor’s active area, enabling the capture of light emissions. Since the BL signal directly correlates with cell viability [258], lensless imaging systems capable of measuring BL can be powerful tools for drug research (toxicity detection) and environmental monitoring [259]. ECL-based lensless imaging involves capturing light emitted from an electrochemical process [104]. This process is driven by an electric field that creates excited states through energetic electron transfer reactions in molecules at electrode surfaces [260]. Unlike CL, which often requires catalysts for significant light emission, ECL reactions utilize the electric potential difference for a more controlled excited state formation. This controlled nature makes ECL advantageous over CL for applications in lensless contact imaging.

### 4.2. Integration of Microfluidic Components with Optical Systems

Microfluidic devices offer precise handling of small volumes of samples, making their application ubiquitous across various areas of biological sciences [261]. A microfluidic device mainly consists of inlets for introducing fluids, outlets for removing fluids, microchannels for guiding, separating, and mixing fluids, and chambers for retaining samples or facilitating reactions [262]. Fluid movement within a microfluidic device can be driven passively through capillary forces within the microchannels or actively using externally applied forces such as pressure from syringe pumps [263]. Additional elements such as valves and flow resistors can be incorporated to regulate the flow within the device.

Lensless image sensors can be integrated with microfluidic devices to facilitate sample manipulation through spatial and temporal control while enabling capabilities such as multiplexing. In this configuration, the microfluidic device is bonded to the lensless image sensor, allowing the region of interest in the microfluidic device to directly contact the active area of the image sensor. Samples and reagents are introduced into the microfluidic device through inlets, with the target analytes within the sample often remaining in chambers that are patterned to physically capture them or functionalized with biorecognition elements to bind them. By surface functionalizing the chamber or adding reaction reagents, analyte-specific reactions can occur in the chamber, enabling analyte quantification by capturing and analyzing the resulting optical signals with the integrated image sensor. This integrated approach originated in 2005 by Lange et al., who placed a microfluidic chamber containing Caenorhabditis elegans on a lensless CMOS image sensor to study the impact of spaceflight on its behavior [264]. Since then, the combination of microfluidic devices and lensless imaging has been adopted in various platforms, enabling the identification, quantification, and monitoring of cells and organisms as well as the detection of biological elements, including metabolites, viruses, and proteins [13,104,105,196].

Integrating microfluidics with image sensors combines the capabilities and advantages of both technologies. The microfluidic component allows for multiplexing through separate channels for different reactions, precise fluid handling with spatial and temporal control, low sample and reagent consumption due to the small volume intake into the device, and compact size. Meanwhile, image sensors enable the quantification of target analytes by measuring the optical changes from the analyte-specific reactions happening in the microfluidic device. The combination results in lensless microfluidic imaging systems that are compact, provide high spatial resolution without compromising the FOV, and produce sensitive results due to the close sample-to-sensor proximity and high photon collection efficiency.

In lensless contact imaging, the proximity between the sample and the image sensor is critical for optimal performance. This technique can be implemented using either shadow-based or luminescence-based methods. Shadow-based imaging benefits from minimal diffraction artifacts and does not require complex image reconstruction, while luminescence-based imaging—including CL, ECL, and BL—provides enhanced optical contrast and quantitative analyte detection. In both cases, maintaining a minimal distance between the sample and the image sensor is essential for achieving high image clarity and maximizing light collection efficiency. To ensure close contact, the design of the microfluidic device must accommodate the topography of the image sensor. This can be challenging due to the size difference between sensor elements and microfluidic channels. However, it can be managed by confining the microfluidic device within the sensor’s active area or by embedding the sensor in a planarizing material to match the fluidic channel dimensions.

## 5. Applications of Optical Image-Based Sensors at Point-of-Care/Need

### 5.1. Applications of Integrated CL Biosensors at Point-of-Care/Need

Integrated optical biosensing technology offers several advantages over traditional microscopy, such as increased compactness, improved photon collection efficiency, and the elimination of complex image reconstruction steps. This technique is versatile, enabling applications like cell detection, classification, counting, and continuous monitoring [265,266,267,268,269]. Blood cell counting is particularly well suited due to the portability and automation benefits compared to traditional methods like microscopy and flow cytometry [266,267,268,270]. For instance, the ePetri platform exemplifies this versatility, allowing continuous monitoring of cells and microorganisms, including pathogen identification, like waterborne parasites and malaria [269,271,272,273].

Biomicrofluidics and optoelectronics further enhance biosensing capabilities by utilizing CL or ECL for the detection and quantification of metabolites, antibodies, proteins, viruses in bodily fluids, and antibiotic residues in food [104,274,275,276]. These platforms hold significant implications for POC diagnostics and disease monitoring, such as uric acid measurement in saliva for gout diagnosis and choline concentration monitoring in blood for cancer and cardiovascular disease. The microfluidic channels in many platforms facilitated both the detection of flowing samples and subpixel spatial resolution by leveraging the sample’s subpixel movements as it passed through the channel [271,277,278,279].

Despite significant advances, optical transducers in fluorescence imaging-based biosensors remain limited by their dependence on specific light sources and the need for dedicated optical filters [280]. However, researchers have developed innovative solutions to address these challenges, such as shifting from fluorescence to chemiluminescence-based biosensors. One notable development is a CCD-based CL biosensor that simultaneously quantifies three targets: alkaline phosphatase, parvovirus B19 DNA, and horseradish peroxidase [256]. This was achieved by placing a transparent microfluidic reaction chip made of PDMS or glass slide in close proximity to a thermoelectrically cooled CCD sensor. A fiber optic taper facilitated the efficient transmission of light between the microfluidic chip and the image sensor [256,281]. Additionally, a photodiode array, consisting of 32 individual photodiodes arranged in a 4 × 8 grid configuration, was explored for its potential application in integrated CL microarray readout [282]. Since LFAs were the first option for point-of-care testing due to their portability and inexpensive components, there have been increased efforts to integrate them with image sensors to create independent yet efficient analytical devices. In this context, aflatoxin B1 and type B fumonisins have been detected in maize samples using a multiplex CL biosensor that integrates a multiplex indirect competitive LFA, enzymatic CL reaction, and lensless CCD image sensor [255]. Furthermore, the LFA-CL system was integrated with a CCD image sensor to detect ovalbumin and collagen. This LFA-CL system exhibited detection limits two orders of magnitude lower than those of a similar colorimetric system [283]. Building upon these advancements, a 3D-printed biosensor employing an LFA with CL detection was designed for the on-site analysis of salivary cortisol in astronauts [284]. This sensor incorporates a sealed microfluidic cartridge activated by buttons and capillary forces. Cortisol detection is achieved using a highly sensitive, cooled CCD camera-based CL reader. Despite these research developments, the commercialization of LFA-CL immunoassays faces challenges due to two key limitations: (1) limited bioreagent stability over time and (2) the need for manual reagent addition during the analytical process. These limitations hinder their ability to meet the demands of end users [285].

Shifting focus from CL-based optical sensors, another study presented a novel bioluminescence ATP detection device utilizing a pixelated CMOS image sensor. This platform effectively assessed ATP levels following environmental swabs from various surfaces, including fast-food restaurant tables, kitchen counters, and personal cell phones. These findings highlight the potential of CMOS devices in the food safety industry, particularly for establishing standardized surface cleanliness protocols [13].

In addition to CL and bioluminescence sensors, ECL-based biosensors have also shown significant promise. One early example described a microfluidic microsystem that combined CMOS technology for high-resolution, direct-contact optical imaging of biochemical analyte reactions [286]. This microsystem utilized a two-layered, soft polymer microfluidic network integrated with a CMOS image sensor featuring a 64 × 128-pixel array to detect both CL and ECL of luminol/H_2_O_2_ reaction [286]. Similarly, a different study described a microfluidic platform integrating luminol/hydrogen peroxide ECL detection with a 5-megapixel CMOS image sensor on a single chip [104]. This design allows both sample manipulation and data acquisition on the same platform. Using a single electrode as the electrochemical transducer and a CMOS chip as the integrated detector, the applicability of this platform for detecting uric acid, a relevant biomarker for gout disease, was demonstrated [104].

Recent research describes advancements in single-electrode ECL (SE-ECL) configurations designed to enhance sensitivity, specificity, and compactness for integration with CMOS image sensors in analyte detection. By manipulating the electrical potentials applied to single electrodes within the electrochemical cell, researchers can selectively excite ECL luminophores to produce distinct emission colors (Figure 3i,j) [105]. This strategy employs a single-electrode design for color-coded ECL reactions, integrates microfluidic techniques for efficient sample handling, and utilizes a cost-effective CMOS image sensor to capture emitted light. These improvements show significant promise for developing miniaturized and highly accurate biosensors suitable for POC diagnostics in biomedical, food safety, and environmental monitoring applications.

Overall, integrated optical biosensing platforms offer powerful tools for live specimen imaging, POC diagnostics, and disease monitoring, with significant potential to impact biomedical research, healthcare, and beyond.

### 5.2. Limitations of Integrated CL Biosensors for Point-of-Care Applications

Integrated optical biosensing platforms offer significant advantages for point-of-care diagnostics, such as portability, cost-effectiveness, and rapid imaging. However, their widespread adoption faces several challenges.

While integrated optical biosensing platforms can achieve reasonable resolution, they typically fall short of traditional lens-based microscopy for resolving fine details. High-resolution imaging requires sophisticated reconstruction algorithms, which can limit practical utility due to their computational intensity. The reliance on advanced algorithms can slow down data processing and necessitate significant computing power. Additionally, these platforms struggle to distinguish objects at different depths due to their relatively shallow depth of field. This limitation makes imaging thick samples or obtaining accurate 3D reconstructions challenging. Sample preparation is also critical, as the technique is sensitive to dust, debris, and bubbles. This meticulous preparation requirement may render integrated optical biosensing platforms unsuitable for certain sample types. Moreover, the field of view is limited due to the nature of the setup, and adjusting magnification is not as straightforward as in traditional microscopy. Despite these challenges, advancements in computational imaging, machine learning, and sample preparation techniques offer promising solutions to overcome these limitations. By addressing these challenges, integrated optical biosensing platforms can significantly enhance their utility for point-of-care applications [34,287,288,289].

The reusability of lensless sensors, such as those utilizing CMOS or CCD imaging chips, is dependent on the specific application and sensor design. Generally, these sensors are designed for multiple uses, though their longevity can be influenced by factors like sensor surface stability, sample nature, and required sensitivity. For example, in the case of chemiluminescence biosensors, it was observed that a CMOS sensor exhibited good reusability, with a low relative standard deviation (RSD) in measurements after five uses, and maintained acceptable performance after ten uses [104]. Additionally, the affordability of CMOS chips, due to advancements in manufacturing, has made them suitable for use as disposable components in single-use biosensors, especially in point-of-care diagnostics. While CCD chips offer higher sensitivity and image quality, they are typically more expensive, and their use as disposable elements is more application-specific, depending on the required sensitivity and cost considerations (Box 2).

Box 2 **Chemiluminescence-based biosensors: types, configurations, and integration.**

 Chemiluminescence (CL) is a phenomenon where light is emitted as a result of a chemical reaction. CL-based biosensors leverage this property to detect and quantify various analytes with high sensitivity and specificity. These biosensors are particularly valued for their ability to produce measurable optical signals without the need for external light sources.

 Chemiluminescence (CL) biosensors include various phenomena, including chemiluminescence, bioluminescence, and electrochemiluminescence (ECL). Chemiluminescence biosensors utilize direct chemical reactions to emit light, while bioluminescence biosensors employ biological molecules to produce light through biochemical reactions, often utilizing ATP assays for detecting cellular activity. Electrochemiluminescence biosensors combine electrochemical and chemiluminescent techniques to trigger light emission. Each type offers unique advantages: chemiluminescence biosensors are simple and highly sensitive, bioluminescence biosensors provide high specificity for detecting biological substances, particularly through ATP assays, and ECL biosensors offer precise control and high sensitivity for a wide range of applications.

ECL systems can be configured in various ways to optimize performance and application:

- *Three Electrode System*: Consists of a working electrode, a reference electrode, and a counter electrode. This configuration allows precise control over the electrochemical environment, enhancing the accuracy of measurements.
- *Bipolar Electrode System*: Utilizes a floating conductor, serving as both the anode and cathode, with the potential of the solution playing a crucial role in driving the redox reactions. This setup is advantageous for miniaturized and portable applications.
- *Single Electrode System*: Relies on a gradient potential over the surface of a single, partially conductive electrode, such as ITO. This configuration simplifies the design while still facilitating effective ECL reactions.

Optical signal detection in CL biosensors is achieved using point detectors and pixelated detectors:

▪ ***Point detectors***, such as photomultiplier tubes (PMT) and avalanche photodiodes (APD), convert light into electrical signals through the photoelectric effect and avalanche multiplication process, respectively, offering high sensitivity for weak light signals.
▪ ***Pixelated detectors***, including charge-coupled devices (CCD) and complementary metal–oxide–semiconductor (CMOS) sensors, use arrays of photodiodes to capture light and produce high-resolution images. CCDs provide excellent sensitivity and image quality, while CMOS sensors are favored for their low power consumption, faster readout speeds, and cost-effectiveness, enhancing their suitability for portable and point-of-care devices.

The integration of CL biosensors with **microfluidic** technologies and CMOS sensors has led to significant advancements. Microfluidic systems, often referred to as lab-on-a-chip (LOC) platforms, enable the miniaturization and automation of assays, reducing sample and reagent volumes and enhancing reaction efficiency. **CMOS image sensors** provide high-resolution imaging, enabling spatially resolved measurements and the simultaneous detection of multiple analytes. The combined system offers compactness, portability, and the potential for point-of-care diagnostic applications.

## 6. Landscape and Outlook of Optical Image Sensors and Machine Learning in Analytical Chemiluminescence Biosensors

The development of biosensors has seen remarkable progress, marked by distinct generations, each introducing significant advancements. The first generation of biosensors utilized natural receptors such as enzymes, antibodies, and cells as biorecognition elements. These elements interacted with target analytes to generate a signal, which was then converted by a transducer into a measurable and identifiable form [290].

In the second generation, the focus shifted towards enhancing the transducer itself. By incorporating nanomaterials to modify the transducer surface, there was a marked improvement in sensitivity and signal amplification. This era also saw the miniaturization of biosensors, making them suitable for point-of-care and portable applications [290,291].

The third generation introduced the capability of simultaneous detection of multiple analytes, achieved through direct signal transfer between transducers and analytes. This advancement significantly improved the specificity and sensitivity of biosensors. The fourth generation further integrated nanotechnology, the Internet of Things (IoT), and bioinspired biomimetic materials [292] with microfluidic platforms. This integration enabled real-time monitoring of biological processes, resulting in instantaneous measurements. Wearable and implantable devices became more prevalent, with lab-on-chip platforms exemplifying this generation [290,291,293].

Looking to the future, the fifth generation of biosensors, illustrated by the concept of a hospital-on-chip, is expected to refine existing technologies even further. The sixth generation will likely incorporate 6G networks and holographic technology into microfluidic systems, potentially leading to innovations such as surgery-on-chip. The Internet of Medical Things (IoMT) will also expand applications in telemedicine [290,291,293].

A rapidly growing field within optical biosensing is quantum sensing, which leverages quantum phenomena like quantum interference, entanglement, and coherence to detect physical properties such as temperature, electromagnetic fields, and strain. Quantum materials can significantly enhance the precision of conventional analytical observations. Numerous quantum biosensors have already been developed for disease diagnosis, and integrating quantum biosensing with fifth- and sixth-generation technologies promises novel and exciting possibilities [291,294].

The future of quantum biosensing holds transformative potential. Anticipated advancements include unprecedented sensitivity, enabling the detection of minute biomarker concentrations, which could revolutionize early disease detection and monitoring, significantly improving patient outcomes. Integrating quantum biosensing with nanotechnology is expected to lead to the miniaturization of devices, enhancing portability and making them suitable for wearable applications. These developments promise a shift towards user-friendly, cost-effective, and rapid POC applications, marking a paradigm shift in disease diagnosis and monitoring [291,294].

The combination of wearable biosensors and ML presents a significant opportunity for health monitoring. Wearable biosensors can noninvasively monitor human physiology through various biological fluids, such as sweat, tears, and saliva. The vision is to integrate a series of sensor networks on flexible patches that continuously monitor biomarkers. ML can parse time-series data from multiplexed sensors to identify health states. However, ML in these applications must be explainable rather than a black-box system. Medical professionals and decision-makers must understand machine decisions, and incorporating human knowledge and reasoning into deep learning systems can enforce and regulate the learning process, reducing the sample size needed for training [295,296,297].

Like other optical biosensors, the development of chemiluminescence biosensors, particularly ECL microscopy, has also shown significant promise. ECL microscopy combines the high sensitivity of electrochemical methods with the spatial resolution of microscopy, making it a powerful tool for visualizing biochemical and cellular processes at a molecular level. This technique has been employed in two experimental configurations: positive ECL (PECL) and shadow ECL (SECL) [298]. In PECL, the imaged object generates ECL directly or is labeled with an ECL-active luminophore, resulting in a bright image against a dark background. SECL, a label-free method, produces a dark image of the object against a bright background by blocking the diffusional flow of the luminophore and co-reactant towards the electrode surface. Both configurations mitigate issues of photobleaching and phototoxicity associated with classical microscopy techniques, contributing to the detailed and precise investigation of various biological systems and reactions. Integrating the advancements in ECL microscopy with innovative technologies in optical biosensing could further enhance the capabilities and applications of biosensors in biomedical research and clinical diagnostics.

The landscape of optical biosensors is rapidly evolving, with each generation bringing significant advancements in technology and functionality. Quantum sensing and machine learning integration are poised to revolutionize biosensing further, enhancing sensitivity, specificity, and usability. These developments herald a future where disease diagnosis and monitoring are more precise, portable, and accessible, paving the way for groundbreaking advancements in healthcare.

Despite these advancements, traditional laboratory assays still outperform POC testing in terms of reliability and accuracy. The integration of machine learning (ML) methods into POCT offers an opportunity to bridge this gap. ML can improve sensor reliability and accuracy in real sample measurements. Smartphone applications integrated with ML algorithms could provide direct readouts of POCT biosensors, potentially pushing these technologies towards home-testing and self-testing scenarios [295].

Single-molecule detection presents significant challenges due to poor signal-to-noise ratios, signal overlap, and dispersive signals. In single-molecule sequencing biosensors, large datasets must be analyzed, and traditional hypothesis-driven data exploration may miss unexpected signals. ML methods can reduce noise and extract multidimensional signal features, improving pattern recognition resolution and sensitivity in single-molecule biosensors [295,299].

Building on these advancements, the application of ML in enhancing CL and ECL biosensors has garnered significant attention. In the following sections, recent progress in integrating ML techniques with ECL and CL biosensors will be reviewed, highlighting how these approaches can improve sensor performance, data analysis, and, ultimately, the practical implementation of these technologies in various testing scenarios.

### 6.1. Machine Learning-Assisted ECL Sensing

Electrochemiluminescence (ECL) sensing offers a powerful technique for detection due to its high signal-to-noise ratio [300]. Traditionally, researchers relied on analyzing ECL data directly. However, recent advancements leverage the power of machine learning (ML) to extract valuable insights from images captured by smartphone cameras. The intensity of light in these images correlates directly with the target analyte’s concentration [301,302].

The field of healthcare diagnostics is a prime beneficiary of this integration. ECL assays are highly sensitive and specific, making them ideal for biomarker detection in body fluids. Early and accurate diagnosis is crucial for effective patient management, and ML-assisted ECL holds significant promise in achieving this goal. Studies have shown that ML techniques can improve detection accuracy in ECL-based assays for various analytes, including glucose, lactate, and choline [303].

Researchers have also explored ML-assisted ECL for environmental and food safety applications. For instance, one study employed a portable molecularly imprinted polymer ECL system with smartphone camera capture and artificial neural networks (ANNs) to detect nitrofurazone, a banned carcinogen [242]. Similarly, another study utilized convolutional neural networks (CNNs) to analyze images from an ECL system designed to detect the harmful pesticide 2,4-Dichlorophenoxyacetic acid (2,4-D) [234]. These examples showcase the versatility of the approach for diverse analytes.

Recent advancements have even extended ML-assisted ECL to the realm of infectious diseases. In response to the COVID-19 pandemic, researchers developed an ML-assisted ECL immunosensor for SARS-CoV-2 detection [304]. This sensor was designed to provide user-friendly readouts comparable to RT-PCR CT values, a familiar metric for healthcare professionals.

Overall, the synergy between ML and ECL sensing is demonstrably powerful. By leveraging image analysis capabilities, this approach offers enhanced accuracy, user-friendliness, and broader applicability across various detection fields, particularly in healthcare diagnostics.

### 6.2. Machine Learning-Assisted Chemiluminescence and Bioluminescence Sensing

Light-emitting luminescence techniques offer valuable tools for analytical detection. Chemiluminescence (CL), where a chemical reaction excites molecules leading to light emission, avoids limitations of fluorescence (FL) like scattering and background noise [305,306]. However, CL suffers from high blank signals and interference from other oxidants and quenchers [307,308].

One study explored CL’s potential for profiling phenolic compounds in wine using a machine learning-assisted assay. Capitalizing on CL’s established role in evaluating food antioxidants, researchers employed neural network regression models (NNRMs) to detect phenolic compounds within a wide concentration range (5 μmol dm^−3^ to 2 mol dm^−3^) [309].

Bioluminescence (BL), a subclass of CL, involves light emission by organisms like fireflies. It offers advantages such as minimal background signal, low required analyte concentration, and high sensitivity due to enzymatic turnover. However, its limitations include dependence on advanced molecular biology techniques for large-scale production of recombinant enzymes [54,310].

While ML-aided BL detection is still less explored compared to other strategies, recent research demonstrates promising applications. A study employed a whole-cell sensing array with genetically modified *E. coli* strains, each expressing a bioluminescence reporter system. This system allowed the detection and classification of various antibiotics based on unique bioluminescence patterns [311]. The patterns were processed into different indices and used to train a multiclass decision forest (MDF) model. This approach achieved over 70% accuracy in classifying eight antibiotics and could even categorize unknown antibiotics based on known samples.

Another study utilized a bioluminescent bacterial system to detect water contaminants. Here, a multilayer perceptron model was trained on light emission kinetics in response to different contaminants, showcasing promising results for rapid detection [312].

Despite the limited number of published studies on ML-assisted CL and BL detection, these examples highlight the exciting potential of this approach for enhancing sensing and biosensing capabilities.

## 7. Conclusions

The integration of optical biosensors into lab-on-a-chip (LOC) platforms represents a significant advancement in the field of analytical biochemistry, enabling precise, real-time detection of various analytes at the point of need. These integrated systems leverage optical phenomena such as fluorescence, chemiluminescence, and electrochemiluminescence to provide high sensitivity and specificity in biomolecular detection. By combining these optical biosensors with microfluidic technologies, it is possible to achieve rapid and efficient sample handling, reducing the overall assay time and minimizing sample volumes.

A crucial development in this field is the coupling of optical biosensors with pixelated image sensors, such as CMOS image sensors, which offer significant advantages over traditional single-point detectors. CMOS image sensors provide high-resolution imaging capabilities, allowing for the simultaneous detection of multiple analytes across a wide field of view. Their pixelated nature enables spatially resolved measurements, which is particularly beneficial for applications requiring detailed mapping and analysis of complex biological samples. Additionally, CMOS sensors are known for their low power consumption, compact size, and cost-effectiveness, making them ideal for integration into portable and point-of-need diagnostic devices.

The development of fully integrated optical biosensors has shown promising results in various applications, including medical diagnostics, environmental monitoring, and food safety. These systems are not only efficient and effective but also pave the way for more widespread and accessible diagnostic solutions.

Looking forward, several exciting developments are anticipated to shape the future of integrated optical biosensors. One of the key areas of growth is the incorporation of machine learning algorithms to enhance the data analysis and interpretation capabilities of these biosensors. Machine learning can facilitate the processing of complex datasets, identify patterns, and provide predictive analytics, thereby improving the accuracy and reliability of biosensor outputs.

Advancements in microfluidic technology are expected to further refine the integration process, allowing for more complex and multifunctional LOC platforms. Innovations in material science and nanotechnology will likely contribute to the development of more sensitive and selective biosensors, expanding their range of detectable analytes and improving their performance in diverse environments.

Additionally, the continued miniaturization and integration of optical components, coupled with advancements in portable power sources and wireless communication technologies, will enable the development of truly portable and user-friendly point-of-need diagnostic devices. These devices will be particularly valuable in resource-limited settings, providing essential diagnostic capabilities without the need for extensive laboratory infrastructure.

In summary, the future of fully integrated optical biosensors holds immense potential, driven by advancements in microfluidics, material science, and machine learning. These innovations will pave the way for more sophisticated, efficient, and accessible diagnostic tools, revolutionizing point-of-need applications across various fields.

## Figures and Tables

**Table 1:** Optical detectors and their characteristics.

Characteristics	PMT	APD	Plate Reader * (PMT)	CCD	CMOS
Photosensitivity (A/W)	0.02–0.09	0.2–0.9	0.02–0.09	0.3–0.4	0.3–0.5
Gain	10^5^–10^7^	10–100	10^5^–10^7^	–––––	–––––
Dynamic Range (dB)	–––––	–––––	–––––	60–90	50–70
Quantum Efficiency (%)	20–35	70–85	20–25	75–90	70–90
Dark Current ** (nA)	0.5–5	0.1–100	0.5–5	10^−10^–10^−8^	10^−8^–10^−7^
Cost	Expensive	Moderate	Expensive	Moderate	Low
Power Consumption	High	Moderate	High	High	Low
Size/Portability	Bulky	Compact	Bulky	Compact	Compact
Multiplexing	No	No	Yes	Yes	Yes
POC	No	Yes	No	Yes	Yes

* Italicized specifications for plate reader are identical to PMT ** CCD and CMOS dark current per pixel calculated from e−/p/s CMOS dark current calculated from DN/s to e−/p/s with conversion factor. –[dash] parameter is not applicable. Specifications for PMT, APD, CCD, and CMOS were sourced from Hamamatsu datasheets. Website: https://www.hamamatsu.com/us/en.html (accessed on 15 July 2024) [217].
